# Aquaculture genomics, genetics and breeding in the United States: current status, challenges, and priorities for future research

**DOI:** 10.1186/s12864-017-3557-1

**Published:** 2017-02-20

**Authors:** The Aquaculture Genomics, Genetics and Breeding Workshop, Hisham Abdelrahman, Mohamed ElHady, Acacia Alcivar-Warren, Standish Allen, Rafet Al-Tobasei, Lisui Bao, Ben Beck, Harvey Blackburn, Brian Bosworth, John Buchanan, Jesse Chappell, William Daniels, Sheng Dong, Rex Dunham, Evan Durland, Ahmed Elaswad, Marta Gomez-Chiarri, Kamal Gosh, Ximing Guo, Perry Hackett, Terry Hanson, Dennis Hedgecock, Tiffany Howard, Leigh Holland, Molly Jackson, Yulin Jin, Karim Kahlil, Thomas Kocher, Tim Leeds, Ning Li, Lauren Lindsey, Shikai Liu, Zhanjiang Liu, Kyle Martin, Romi Novriadi, Ramjie Odin, Yniv Palti, Eric Peatman, Dina Proestou, Guyu Qin, Benjamin Reading, Caird Rexroad, Steven Roberts, Mohamed Salem, Andrew Severin, Huitong Shi, Craig Shoemaker, Sheila Stiles, Suxu Tan, Kathy F. J. Tang, Wilawan Thongda, Terrence Tiersch, Joseph Tomasso, Wendy Tri Prabowo, Roger Vallejo, Hein van der Steen, Khoi Vo, Geoff Waldbieser, Hanping Wang, Xiaozhu Wang, Jianhai Xiang, Yujia Yang, Roger Yant, Zihao Yuan, Qifan Zeng, Tao Zhou

**Affiliations:** 1 School of Fisheries, Aquaculture and Aquatic Sciences, Auburn University, Auburn, AL 36849 USA; 2 Department of Biological Sciences, Auburn University, Auburn, AL 36849, USA; 3 Environmental Genomics Inc., P. O. Box 196, Southborough, MA 01772-1801 USA; 4 Aquaculture Genetics & Breeding Technology Center, Virginia Institute of Marine Science, Gloucester Point, VA 23062 USA; 5 Department of Biology, Middle Tennessee State University, Murfreesboro, TN 37132 USA; 6 Aquatic Animal Health Research Unit, USDA-ARS, 990 Wire Road, Auburn, AL 36832 USA; 7 USDA-ARS-NL Wheat & Corn Collections at a Glance GRP, National Animal Germplasm Program, 1111 S. Mason St., Fort Collins, CO 80521-4500 USA; 8 USDA-ARS/CGRU, 141 Experimental Station Road, Stoneville, MS 38701 USA; 9 Center for Aquaculture Technologies, 8395 Camino Santa Fe, Suite E, San Diego, CA 92121 USA; 10 Department of Fisheries and Wildlife, Oregon State University, Corvallis, OR 97331 USA; 11 Department of Fisheries, Animal & Veterinary Science, 134 Woodward Hall, 9 East Alumni Avenue, Kingston, RI 02881 USA; 12 Haskin Shellfish Research Laboratory, Department of Marine and Coastal Sciences, Rutgers University, 6959 Miller Avenue, Port Norris, NJ 08349 USA; 13 Department of Genetics, Cell Biology and Development, 5-108 MCB, 420 Washington Avenue SE, Minneapolis, MN 55455 USA; 14 Department of Biological Sciences, University of Southern California, Los Angeles, CA 90089-0371 USA; 15 Taylor Shellfish Farms, 130 SE Lynch RD, Shelton, WA 98584 USA; 16 Department of Biology, University of Maryland, 2132 Biosciences Research Building, College Park, MD 20742 USA; 17 National Center for Cool and Cold Water Aquaculture, Agricultural Research Service, United States Department of Agriculture, Kearneysville, WV 25430 USA; 18 Troutlodge, 27090 Us Highway 12, Naches, WA 98937 USA; 19 USDA ARS NEA NCWMAC Shellfish Genetics at the University Rhode Island, 469 CBLS, 120 Flagg Road, Kingston, RI 02881 USA; 20 Department of Applied Ecology, North Carolina State University, Raleigh, NC 27695-7617 USA; 21 USDA ARS Office of National Programs, George Washington Carver Center Room 4-2106, 5601 Sunnyside Avenue, Beltsville, MD 20705 USA; 22 School of Aquatic and Fishery Sciences, University of Washington, Seattle, WA 98105 USA; 23 Genome Informatics Facility, Office of Biotechnology, Iowa State University, Ames, IA 50011 USA; 24 USDOC/NOAA, National Marine Fisheries Service, NEFSC, Milford Laboratory, Milford, Connectcut 06460 USA; 25 School of Animal and Comparative Biomedical Sciences, University of Arizona, Tucson, AZ 85721 USA; 26 Aquatic Germplasm and Genetic Resources Center, School of Renewable Natural Resources, Louisiana State University Agricultural Center, Baton Rouge, LA 70820 USA; 27 Stonebridge breeding Ltd, Gate House, Abbotswood, Evesham, WR11 4NS UK; 28 Aquaculture Genetics and Breeding Laboratory, The Ohio State University South Centers, Piketon, OH 45661 USA; 29 Key Laboratory of Experimental Marine Biology, Institute of Oceanology, Chinese Academy of Sciences, Qingdao, 266071 China; 30 Hybrid Catfish Company, 1233 Montgomery Drive, Inverness, MS 38753 USA

**Keywords:** Aquaculture, Genetic resources, Genome, Transcriptome, QTL, RNA-Seq, SNP, Fish, Shellfish

## Abstract

Advancing the production efficiency and profitability of aquaculture is dependent upon the ability to utilize a diverse array of genetic resources. The ultimate goals of aquaculture genomics, genetics and breeding research are to enhance aquaculture production efficiency, sustainability, product quality, and profitability in support of the commercial sector and for the benefit of consumers. In order to achieve these goals, it is important to understand the genomic structure and organization of aquaculture species, and their genomic and phenomic variations, as well as the genetic basis of traits and their interrelationships. In addition, it is also important to understand the mechanisms of regulation and evolutionary conservation at the levels of genome, transcriptome, proteome, epigenome, and systems biology. With genomic information and information between the genomes and phenomes, technologies for marker/causal mutation-assisted selection, genome selection, and genome editing can be developed for applications in aquaculture. A set of genomic tools and resources must be made available including reference genome sequences and their annotations (including coding and non-coding regulatory elements), genome-wide polymorphic markers, efficient genotyping platforms, high-density and high-resolution linkage maps, and transcriptome resources including non-coding transcripts. Genomic and genetic control of important performance and production traits, such as disease resistance, feed conversion efficiency, growth rate, processing yield, behaviour, reproductive characteristics, and tolerance to environmental stressors like low dissolved oxygen, high or low water temperature and salinity, must be understood. QTL need to be identified, validated across strains, lines and populations, and their mechanisms of control understood. Causal gene(s) need to be identified. Genetic and epigenetic regulation of important aquaculture traits need to be determined, and technologies for marker-assisted selection, causal gene/mutation-assisted selection, genome selection, and genome editing using CRISPR and other technologies must be developed, demonstrated with applicability, and application to aquaculture industries.

Major progress has been made in aquaculture genomics for dozens of fish and shellfish species including the development of genetic linkage maps, physical maps, microarrays, single nucleotide polymorphism (SNP) arrays, transcriptome databases and various stages of genome reference sequences. This paper provides a general review of the current status, challenges and future research needs of aquaculture genomics, genetics, and breeding, with a focus on major aquaculture species in the United States: catfish, rainbow trout, Atlantic salmon, tilapia, striped bass, oysters, and shrimp. While the overall research priorities and the practical goals are similar across various aquaculture species, the current status in each species should dictate the next priority areas within the species. This paper is an output of the USDA Workshop for Aquaculture Genomics, Genetics, and Breeding held in late March 2016 in Auburn, Alabama, with participants from all parts of the United States.

## Background

The major goals of research programs having components related to aquaculture genomics, genetics and breeding are to enhance aquaculture production efficiency, sustainability, product quality and profitability in support of the commercial sector and for the benefit of U.S. consumers. Progress towards achieving these goals includes genetic improvement of production, performance and animal welfare/fitness traits, and this progress is predicated upon the access and utilization of an array of genetic resources within each species group. To this end, various genetic stock enhancement approaches are currently being studied by the aquaculture research community, and major progress has been made since the start of aquaculture genomics research 20 years ago [1]. Such progress includes advances in traditional selection, intraspecific crossbreeding, interspecific hybridization, genome-enabled selection (e.g., marker/causal mutation-assisted selection and/or genomic selection), polypoidy, sex reversal and breeding, xenogenesis, gene transfer, and genome editing. Some of the most important traits studied for genetic improvement in U.S. aquaculture species include disease resistance, feed conversion efficiency, growth rate, behaviour, processing yield, reproductive characteristics and tolerance to environmental stressors like low dissolved oxygen, high or low water temperature and salinity, body composition, and flesh quality. Traditionally, genetic improvement in the commercial aquaculture sector relied on phenotypes and pedigree information, but recently leading international breeding companies have begun to implement genome technologies into their breeding programs for some of the species where advanced genomic resources and tools are available (e.g., [2–4]).

Genomic information provides powerful tools to enhance physiological research, the results of which may be used for optimization of husbandry practices, feeding and feed formulations, breeding technologies, or non-genetic selection or screening (e.g., epigenetics, proteomics, and metabolomics). Whole genome sequences, in various states of assembly, are now available for many aquaculture species, enabling the identification of genomic variations such as insertions/deletions, single nucleotide polymorphisms (SNPs), copy number variations, and differentially methylated regions. However, this information is only useful when used to predict phenotypes that have a positive impact on production or product quality. For this reason, genetic mapping, quantitative trait loci (QTL) analysis, genome-wide association studies (GWAS), expression profiling, and bioinformatic analysis can be used to identify genotypic variants associated with particular phenotypic traits, which can then be exploited in breeding programs. For some aquaculture species we have reached the point where genome-based technologies such as marker-assisted and whole genome selection can be applied to enhance aquaculture traits and research is beginning to shift toward understanding functional polymorphisms and the gene regulatory networks underlying commercially important traits. A more complete understanding of the gene networks underlying growth, reproduction, and disease resistance will provide the knowledge-base for developing more robust and productive genetic stocks for the aquaculture industry.

The degree to which genome enabled technologies and genomic information have been or can be applied in genetic improvement programs varies across aquaculture species. Private sector investment in research and development for the implementation of new technologies is dependent on unique industry structure (e.g., overall size of the industry, size of individual companies) and the level of vertical integration. In addition, the approach used for germplasm improvement and status of existing breeding programs dictates whether and which genome enabled technologies are suitable for a given industry. Industries with centralized breeding, such as rainbow trout and salmon, have greater potential to benefit from new technologies compared to industries where breeding activities are widely distributed. Finally, the current demand for species-specific genomic tools (such as high through-put genotyping assays) among the diverse aquaculture industry sectors is low, rendering them commercially unaffordable. This forces some industries interested in genetic improvement to rely on the public sector for resources that enable application of state-of-the-art genomic technologies.

Here we review the development of genomic tools and application of genome enabled technologies for the genetic improvement of aquaculture species. Specifically, we review the status of genome mapping and sequencing, identify gaps in our current knowledge, and highlight the need to implement new technologies in aquaculture. We then propose a set of priorities for future research in aquaculture genomics, genetics, and breeding.

### Whole genome sequencing and assembly

The genomes of several major aquaculture species in the United States, especially those under the USDA National Research Support Project 8 (NRSP-8), have been sequenced or are being sequenced (Table 1), including catfish [5], Atlantic salmon [6], rainbow trout [7], tilapia [8], striped bass (Reading, personal communication), Pacific oyster [9], eastern oyster (Gomez-Chiarri, personal communication), and Pacific white shrimp (Xiang, personal communication) as well as yellow perch and bluegill sunfish (Wang, personal communication). These accomplishments were achieved through support of USDA, NOAA, and other U.S. funding agencies. National Institute of Food and Agriculture (NIFA) AFRI programs, especially the Animal Genomics, Genetics and Breeding program, were central to the historical achievements of generating the reference genome sequences for these fish and shellfish species. Strong international collaboration was also important for the achievements. For instance, the genome project for the Pacific oyster was led by scientists from China and the U.S. [9]; the Atlantic salmon project was led by scientists from Norway, Canada, and Chile [6]; the rainbow trout project was led by scientists from France and currently is a collaborative international effort primarily between the U.S. and Norway; the genome project for Pacific white shrimp is led by Chinese scientists (Xiang, personal communication), and a reference genome for the original specific pathogen-free (SPF) broodstocks developed by the U.S. Marine Shrimp Farming Program in Oahu, HI is being generated (Alcivar-Warren, personal communication).

**Table 1:** Some examples of whole genome sequencing of aquatic and aquaculture species

Species	References
***Ictalurus punctatus*** **(Channel catfish)**	**Liu et al. 2016** [5]
***Ictalurus furcatus*** **(Blue catfish)**	**Waldbieser and Liu, unpublished data**
**Oncorhynchus mykiss (Rainbow trout)**	**Berthelot et al. 2014** [7]
***Salmo salar*** **(Atlantic salmon)**	**Lien et al. 2016** [6]
***Oreochromis niloticus*** **(Nile tilapia)**	**Brawand et al. 2014** [8]
***Crassostrea virginica*** **(Eastern oyster)**	**Gomez-Chiarri et al. 2015** [15] & **personal communication**
***Crassostrea gigas*** **(Pacific oyster)**	**Zhang et al. 2012** [9]
***Penaeus/Litopenaeus vannamei*** **(Pacific white shrimp)**	**Xiang, 2016, personal communication**
***Penaeus monodon*** **(Giant tiger prawn)**	**Warren, personal communication**
Atlantic Cod	Star et al. 2011 [142]
Bluegill sunfish	Wang, personal communication
California yellowtail	Severin, Purcell, Hyde, personal communication
Cavefish	McGaugh et al. 2014 [143]
Coelacanth	Amemiya et al. 2013 [144]
Common carp	Xu et al. 2014b [145]
Indian catfish	Das, personal communication
Japanse flounder	Chen, Yellow Sea Fisheries Institute, China, personal communication
Grass carp	Wang et al. 2015 [146]
Lamprey	Smith et al. 2013 [147]
Medaka	Kasahara et al. 2007 [148]
Pacific abalone	Severin, Purcell, Hyde, personal communication
Pearl oyster	Du, personal communication
Platyfish	Schartl et al. 2013 [149]
Rohu carp	Das, personal communication
Sea bass	Tine et al. 2014 [150]
Scallops	Bao, Ocean University of China, personal communication
Sea cucumber	Xiang, Chinese Academy of Sciences, China, personal communication
Shark	Venkatesh et al. 2014 [151]
Sole	Chen et al. 2014 [152]
Stickleback	Jones et al. 2012 [153]
Striped bass	Reading, 2016, personal communication
Tetraodon	Jaillon et al. 2004 [154]
Turbot	Figueras et al. 2016 [155]
White bass	Reading, 2016, personal communication
Yellow croaker	Wu et al. 2014 [156]
Yellow perch	Wang, personal communication
Zebrafish	Howe et al. 2013 [157]

Bold data are the species initially included in the NRSP-8 Project (Alcivar-Warren et al. 1997)

Various technologies have been used for the generation of the whole genome sequence of aquaculture species. However, the Illumina and PacBio platforms have contributed the most to the progress of aquaculture genome sequencing. Illumina sequencing generates accurate, but short reads at a relatively low cost, while PacBio sequencing generates longer, but less accurate reads at a higher cost. The proportion of sequences generated using these two platforms varies depending on the species and the status of the sequencing technology when the genome sequencing projects were initiated. A significant decrease in the cost of PacBio sequencing has generally led to increased use of this technology and the enhancement of contig/scaffold lengths in sequence assemblies.

Like the selection of sequencing technologies, various sequencing templates were used for the generation of the whole genome sequence assemblies in aquaculture species. These included mixtures of outbred individuals, single diploid males or females, individuals from inbred lines, and completely homozygous doubled haploids. The use of more homozygous templates greatly simplifies the computation of genome assemblies, which are complicated by the high levels of heterozygosity and sequence polymorphism characteristic of several aquaculture species [6, 7, 10]. The choice of sequencing templates has largely been dictated by the availability of the preferred homozygous templates. For instance, doubled haploids, produced through gynogenesis or androgenesis, are the preferred sequencing template for most teleost sequencing projects. However, the generation of doubled haploids is not generally feasible in many shellfish species, due to the unequal first cleavage that is sensitive to manipulation [11]. For some species, multiple individuals must be used because the DNA extracted from a single individual is not sufficient for the sequencing process.

While the mechanics of generating a large number of sequence reads is no longer difficult, calculation of a high quality of sequence assembly remains a challenging task. Four specific metrics are generally used to evaluate the quality of whole genome sequence assemblies including 1) *Contiguity*, as reflected in contig numbers and distribution of contig sizes; 2) *Connectivity*, as reflected in the number of scaffolds and distribution of scaffold sizes; 3) *Completeness*, as reflected in the total size of the genome assemblies and the percentage of coverage of the whole genome; and 4) *Accuracy*, as validated by at least one additional methodology such as genetic linkage mapping, physical mapping, or optical mapping. In addition, the integration of the whole genome sequences with genetic linkage maps is important for genetic studies.

The quality of current whole genome sequences as measured by these four metrics, varies among species. Quality measurements of the whole genome sequence assemblies of the aquaculture species are summarized in Table 2. In general, sequence assemblies of fish species are of higher quality than those for shellfish species. This is in part because the genomes of the shellfish species are highly heterozygous and contain a high level of repetitive elements. For instance, the oysters are among the most polymorphic animals; SNP density was estimated at 1.22 SNPs per 100 bp for the Pacific oyster [9] and either 1.85 SNPs [12] or 4.2 SNPs per 100 bp [10] at population levels for the eastern oyster. Moreover, repetitive elements account for over 80% of the shrimp genome (Xiang, personal communications).

**Table 2:** Status of whole genome sequencing and assembly of major aquaculture species in the United States, listed in the order of scaffold N50 sizes

Species	Contig N50 [141]	Scaffold N50 (Mb)	Scaffolds	% on chromosome	Sequencing platform	Total size (Mb)	References
Catfish	77.2	7.73	9974	97.2	Illumina, PacBio	783	Liu et al. 2016 [5]
				99.1			Zeng et al. 2017 [13]
Atlantic Salmon	57.6	2.97	843,055	75.4	Sanger, Illumina, PacBio	2970	Lien et al. 2016 [6]
Tilapia	29.3	2.80	-	70.9	Illumina, PacBio	928	Brawand et al. 2014 [8]
	3090	-		86.9		1010	Conte et al. 2016, PC
Eastern oyster	1.59	2.50	849	In progress	PacBio, Illumina	819	Wes Warren, PC
Rainbow trout	7.7	0.38		54.0	Illumina	1900	Brawand et al. 2014 [8] Palti and Gao, PC
	13.9	1.72		82.0		2178	
Zebrafish	25.0	1.55		96.5	Sanger, Illumina	1410	Howe et al. 2013 [157]
California yellowtail	139.3	1.49	4439	-	Illumina	685	Andrew Severin, PC
					PacBio		
Pacific white shrimp (Litopenaeus vannamei)	57.1	0.66	6007	71.6	Illumina	1779	Jianhai Xiang, 2016, PC
					PacBio		
Pacific oyster	19.4	0.4	11,969	-	Illumina	559	Zhang et al. 2012b [9]
Striped bass	20.9	0.03	35,010	-	Illumina	585	Benjamin Reading, 2016, PC
					PacBio		
White bass (male/female)	In process	In process	56,818/57,533	-	Illumina	644/643	Benjamin Reading, 2016, PC
Pacific abalone	In process	In process	-	-	Illumina	2000	Severin, Purcell, Hyde, PC
Yellow perch (male/female)	In process	In process	-	-	Illumina	1380/1240	Haping Wang, PC

Zebrafish is included as a reference. PC: personal communications

For species under the NRSP-8 program, reference genome sequence assemblies for catfish, tilapia, Atlantic salmon, and rainbow trout are of good quality. For catfish, 50% of the genome sequence is included in only 31 of the largest scaffolds; 90, 95, and 98% of the genome is included in 185, 314, and 594 scaffolds, respectively. The catfish reference genome sequence was assessed to be nearly complete as 99.7% of re-sequencing reads were mapped to the reference genome sequence. In addition, the number of complete genes included in the reference genome sequence is larger than that of any of the sequenced diploid fish species, including zebrafish [5]. The catfish reference genome sequence assembly was validated by genetic mapping. The positions of 253,744 genetically mapped SNPs were fully concordant with those on the reference genome sequence with four exceptions [13]. The vast majority of the reference genome sequence (99.1%) has been anchored to chromosomes [13].

The reference genome assembly of Atlantic salmon is also of high quality [6]. The genome was sequenced with Sanger and Illumina technologies. It is complete as 2.97 Gb reference genome sequences were assembled, with the unassembled sequences being just repetitive elements. The largest 9447 scaffolds accounted for 2.24 Gb of the 2.97 Gb genome sequence. This is a remarkable achievement considering the very complex nature of the genome. The Atlantic salmon genome is largely tetraploid due to a recent genome duplication. It also has a high repeat content (58–60%); the dispersed Tc1 transposons represented 12.89% of the genome [6]. Similarly, the assembly of the rainbow trout genome is of good quality [7]. Since the publication of the genome paper, the reference genome sequence of rainbow trout has been further improved. The contig N50 has increased from 7.7 Kb to 13.9 Kb, and the scaffold N50 has increased from 380 Kb to 1.72 Mb. More importantly, over 82% of the genome sequence has been mapped to chromosomes (Palti, personal communication).

The published tilapia genome sequence [8] was already of good quality, but the recent use of PacBio long sequencing technology allowed a new high quality assembly (Matthew Conte, personal communication). The contig L50 length reached 3.09 Mb, and 50% of the genome is included in the largest 93 contigs. Importantly, over 86.9% of the reference genome sequence is anchored to chromosomes, enhancing the utility of the reference genome sequence for genetic analyses. The whole genome sequences of striped bass, white bass, yellow perch, and bluegill sunfish are at the stage of draft assemblies.

A published genome sequence exists for the Pacific oyster, but the assembly is highly fragmented [9]. Efforts are ongoing to improve the genome assembly and contiguity, completeness, and accuracy are significantly better now (Zhang, personal communication). Linkage analyses were conducted to validate the genome sequence assembly [14]. The whole genome assembly of eastern oysters is at the draft sequence stage. Several strategies were employed to address challenges encountered in the assembly of the Pacific oyster genome. A single, highly inbred individual, produced through multiple generations of inbreeding and one generation of meiotic gynogenesis (Guo, personal communication) was used as a template. PacBio sequencing was used to provide 50x genome coverage in addition to Illumina sequencing ([15]; Gomez-Chiarri, personal communication). Initial statistics suggest this assembly is of much higher quality than that of the Pacific oyster. The draft assembly (Table 2) is now being validated using high-density linkage maps generated by the Guo laboratory.

The Pacific shrimp genome has been sequenced and assembled, but is not yet published. As shown in Table 2, the genome assembly is of high quality, with a contig N50 of 57.1 kb. The whole genome is included in 6007 scaffolds. Importantly, 71.6% of the genome sequence is anchored to chromosomes through linkage mapping (Xiang, personal communications). Of all the aquaculture genomes, the shrimp genome is perhaps the hardest to deal with because of the difficulty in isolating high molecular weight DNA due to enhanced DNase activity, the large chromosome number, and high levels of heterozygosity and repetitive elements. Physical mapping has been hindered by the lack of BAC libraries with very large inserts. The only BAC library of shrimp, pECBAC1, has an average insert size of approximately 101 kb [16].

For XY heterogametic species, often only the homogametic gender was used as sequencing template, and so information on the sex chromosomes is lacking. For instance, the catfish genome sequence was produced using a doubled haploid female produced through gynogenesis, and therefore the Y sex chromosome was not sequenced. Similarly, the Atlantic salmon genome was produced by using DNA template from a single double-haploid female produced by mitotic androgenesis. Therefore, the Y chromosome is not included in the reference genome. The rainbow trout genome was sequenced using a YY doubled haploid. While it provided Y chromosome information, the X chromosome was not covered in the reference genome sequence. Furthermore, sex determination in some fish and shellfish is complicated by having multifactorial sex determining mechanisms, including genetic sex determination (GSD), environmental sex determination (ESD) and their interactions. With WZ heterogametic species like some of tilapia species, sequencing a single representative of each gender may not be sufficient if there is a polygenic sex determination.

The first genome sequence is a historical milestone for any aquaculture species. However, in order to enable the utility of a reference sequence, additional work is required. For all aquaculture species, further refinement of the reference genome sequence, including improvements in contiguity, completion, and accuracy, as well as anchoring the reference genome sequence to chromosomes and obtaining sex chromosome sequences, is a priority (Table 3). Integration of genome sequence and linkage maps is also very important for genetic and breeding work, and can be accomplished relatively quickly. Sequencing of the Y or X chromosome is essential to study sex determining mechanisms, and sex-related traits, such as sexual dimorphism in growth or sexual size dimorphism (SSD). For instance, with tilapia and bluegill, males grow much faster and bigger than females. In contrast, females grow faster and bigger with yellow perch (Hanping Wang, personal communication). Such differences can be exploited as excellent natural models for the analysis of the genomic basis for sexual bimorphisms.

**Table 3:** Examples of additional work to enhance the utility of the whole genome reference sequences of major aquaculture species in the United States

Species	Contiguity, completion, and accuracy	Anchoring sequence to chromosomes	Sex chromosome sequencing
Catfish	+	+	Y chromosome need to be sequenced
Atlantic salmon	++	++	Y chromosome need to be sequenced
Tilapia	+	+	
Rainbow trout	++	+++	
California yellowtail	++	+++++	
Pacific oyster	+++	+++	
Striped bass	++++	+++++	
White bass	++++	+++++	
Eastern oyster	+++	++++	
Shrimp	+++	+++	
Pacific abalone	+++	+++++	

+ indicate some additional work required, and additional “+” signs indicate the level of additional work required; additional “+” signs indicate larger amount of improvements are needed

### Genomic variations, polymorphic markers, and genotyping platforms

Catalogues of genome variations and efficient genotyping platforms are essential to fully exploit whole genome sequences. One of the most useful by-products of whole genome sequencing is the development of thousands of DNA markers. In the first decade of aquaculture genome research, major effort was focused on developing polymorphic markers [17]. As whole genome sequencing projects were conducted, large numbers of polymorphic markers were identified. Whole genome sequencing with diploid sequencing templates allows identification of both microsatellites and SNPs. Analysis of SNPs between the two alleles of the sequenced individual also allow a rough assessment of the level of heterozygosity of the species.

In addition to whole genome sequencing, SNPs can be identified through genome re-sequencing or RNA-Seq projects. For instance, genome re-sequencing projects have identified more than 8.3 and 9.7 million putative SNPs in channel catfish [18] and Atlantic salmon [19] respectively. Large numbers of SNPs have been identified in most major aquaculture species, with those for the species under the NRSP-8 summarized in Table 4. SNP markers are a much-needed resource for genetic and genomic studies, the construction of high-density SNP arrays, and the development of high-density linkage maps. Validation and testing of these SNPs using SNP arrays will form the material basis for GWAS and whole genome-based selection.

**Table 4:** Some examples of SNPs identified from the aquaculture species under NRSP-8

Species	SNPs from genome sequencing	Numbers of SNPs	Method of identification	Reference
Catfish	None	8.3 million	Genome re-sequencing, transcriptome sequencing	Sun et al. 2014 [18]
				Liu et al. 2012 [158]
Rainbow trout	None	145,168	RAD sequencing	Palti et al. 2014 [159]
		5052	RNA-Seq	Christensen et al. 2013 [160], Al-Tobasei et al. 2016 [161]
		50,000	RNA-Seq	Palti et al. 2015 [23]
		1.8 million	Genome re-sequencing	
Atlantic salmon	None	9.7 million	Genome re-sequencing	Yáñez et al. 2016 [19]
Tilapia	Yes	3569	Genome re-sequencing	Van Bers et al. 2012 [162]
Striped bass	Yes	-	RNA-Seq	Li et al. 2014 [163]
Pacific oyster	Yes	3.8 million	Genome re-sequencing	Zhang et al., 2012 [9]
		4122	RNA-Seq	Hedgecock et al. 2015 [14]
Pacific white shrimp	Yes	96,040	RNA-Seq	Yu et al. 2014 [164]

Those SNPs identified from genome sequencing are not included here

A key advantage of SNP over microsatellite markers is the potential for rapid, low-cost genotyping. For many aquaculture species, the identification of large numbers of SNPs led to the development of efficient genotyping platforms. Available high-density SNP arrays for aquaculture species are listed in Table 5 and include the 15, 286, and 930 K Atlantic salmon arrays [6, 20, 21], the 250 and 690 K catfish arrays [13, 22], the 57 K rainbow trout array [23], and the 250 K common carp array [24]. The SNP arrays for each of the four aforementioned species have high marker densities and good genome coverage. SNP arrays need to be developed for tilapia, striped bass, oysters, and shrimp. As with genome assembly, the development of SNP arrays for some species (e.g., oysters and shrimp) is complicated by extremely high levels of polymorphism.

**Table 5:** Development of high density SNP arrays in aquaculture species, PC: personal communications

Species	SNP array technology	SNP array density	References
Atlantic salmon	Illumina iSelect technology	15 K	Gidskehaug et al. 2011 [20]
	Affymetrix Axiom technology	286 K	Houston et al. 2014 [21]
	Affymetrix Axiom technology	930 K	Lien et al. 2016 [6]
Catfish	Affymetrix Axiom technology	250 K	Liu et al. 2014 [22]
	Affymetrix Axiom technology	690 K	Zeng et al. 2017 [13]
Common carp	Affymetrix Axiom technology	250 K	Xu et al. 2014 [147]
Rainbow trout	Affymetrix Axiom technology	57 K	Palti et al. 2015 [23]
	Affymetrix Axiom technology	50 K	Salem et al. PC

### Linkage mapping and physical mapping

Ultimately, genomic information must be translated into genetic terms to facilitate genetic enhancement in aquaculture. Genetic linkage maps derived from genetic analysis of recombination during meiosis are important for the assembly of chromosome-scale sequence scaffolds. Mapping of sequence-tagged genetic markers derived from the reference genome allows sequence contigs to be arranged in an order that corresponds to the linkage group or chromosome. In addition, linkage mapping is a good method for validating reference genome assemblies.

Linkage maps have been constructed for most of the major aquaculture species (Table 6). For the species under NRSP-8, high density maps exist for catfish, Atlantic salmon, rainbow trout, tilapia, oysters, and shrimp. The linkage maps for catfish and salmonids have the highest marker densities, with the latest catfish linkage map ordering 253,087 markers [13], and the Atlantic salmon linkage map ordering 565,887 markers [6]. The latest linkage maps for the Pacific and eastern oysters have 3367 and 4316 markers, respectively [25] (Guo, personal communication). A large proportion of the genome sequence has been anchored to linkage maps in catfish (99.1%), tilapia (86.9%), Atlantic salmon (75.4%), Pacific shrimp (71.6%), and rainbow trout (54%).

**Table 6:** Examples of genetic linkage maps in aquaculture species, with the species under the NRSP-8 in bold

Species	Number and type of markers	Mapping population	Unique map positions	References
Asian seabass	790 microsatellites and SNPs	93 fish from two families	501	Wang et al. 2011 [165]
**Atlantic salmon**	**5650 SNPs**	**3297 fish from 143 families**	**2894 in female genetic map, 1009 in male specific map**	**Lien et al. 2011** [166]
**Brown trout**	**288 microsatellites, 13 allozymes**	**93 fish from 4 families**	**-**	**Gharbi et al. 2006** [167]
**Catfish**	**54,342 SNPs**	**576 fish from three channel catfish families**	**15,598**	**Li et al. 2015** [168]
	**26,239 SNPs**	**288 interspecific backcross progenies**	**12,776**	**Liu et al. 2016** [169]
	**253,087 SNPs**	**465 fish from four channel catfish families**	**30,591**	**Zeng et al. 2017** [13]
Common carp	28,194 SNPs	108 fish from one yellow river carp family	14,146	Peng et al. 2016 [170]
**Eastern oyster**	**4607 SNPs**	**112 progenies from one family**	**4136**	**Guo, personal communication**
European seabass	190 microsatellites, 176 AFLP, 2 SNP	50 fish from one Venezia Fbis family	-	Chistiakov et al. 2008 [171]
Grass carp	279 microsatellites and SNPs	192 progenies from two families	245	Xia et al. 2010 [172]
Japanese flounder	1268 microsatellites, 105 SNPs, 2 genes	45 offspring from one family	235 in male genetic map, 184 in female genetic map	Castaño-Sánchez et al. 2010 [173]
**Pacific oyster**	**1172 SNPs and microsatellites**	**336 progenies from five families**	**1172 unique markers mapped**	**Hedgecock et al. 2015** [14]
			**424 in consensus linkage map**	
**Rainbow trout**	**2226 microsatellites and SNPs**	**120 individuals from two unrelated doubled haploid lines**	**1366 in synthetic map**	**Guyomard et al. 2012** [174]
	**47,939 SNPs**	**5716 fish**	**47,939 mapped to genome sequence scaffolds**	**Gonzalez-Pena et al. 2016** [26]**; Palti personal communication**
Scallop	3806 SNPs	96 progenies from one Farrer’s scallop family	2983	Jiao et al. 2013 [175]
Sea bream	321 microsatellites, ESTs, and SNPs	50 individuals from one family	229	Tsigenopoulos et al. 2014 [176]
**Giant tiger prawn**	**3959 SNPs**	**1024 offspring from seven black tiger shrimp family**	**-**	**Baranski et al. 2014** [177]
**Pacific white shrimp**	**429 AFLP, 22 microsatellites**	**F2 cross of slow and fast growth parents, 43 shrimp**	**-**	**Andriantahina et al. 2013** [178]
	**6146 SNPs**	**205 progenies from one Pacific white shrimp family**	**4650**	**Yu et al. 2015** [179]
**Tilapia**	**525 microsatellites, 20 genes**	**70 individuals from one family**	**435**	**Lee et al. 2005** [180]
	**401 microsatellites**	**95 individuals from two families**	**352**	**Liu et al. 2013** [181]
Yellowtail	217 microsatellites	90 progenies from one family	105 in female genetic map, 83 in male genetic map	Ohara et al. 2005 [182]
	1480 microsatellites and 601 SNPs	94 offspring of one family	-	Aoki et al. 2015 [183]
	6275 SNPs	460 individuals from five wild families	-	Ozaki et al. 2016 [184]

The major issue for linkage maps of aquaculture species is resolution. While the number of markers on the high density SNP arrays is large, map resolution has been limited by the size of the mapping populations. In most cases, the number of samples used for genetic mapping was not very large, leading to a high level of marker stacking. The exceptions are the Atlantic salmon and rainbow trout where over 2000 and 5000 individuals, respectively, were used for linkage analysis, leading to a very high resolution of the linkage map [6, 26]. While the high fecundity of fish and shellfish species makes it possible to generate large mapping families, the major limitation for high resolution linkage mapping is funding, as genotyping costs are directly proportional to the sample sizes in linkage analysis.

Physical maps have been constructed for only a few aquaculture species (Table 7) including Atlantic salmon [27], tilapia [28], catfish [29, 30], rainbow trout [31], common carp [32], Asian seabass [33], Pacific oyster [34] and scallop [35]. Over time, BAC-based physical mapping has been replaced in favour of next generation sequencing and optical mapping technologies [36]. The existing physical maps and related BAC resources, however, are still useful for validation of reference genome sequences.

**Table 7:** Examples of physical maps constructed from aquaculture species

Species with physical maps	References
Atlantic salmon	Ng et al. 2005 [27]
Tilapia	Katagiri et al. 2005 [28]
Channel catfish	Xu et al. 2007 [30]
	Quiniou et al. 2007 [29]
Pacific oyster	Gaffney, 2008 [34]
Rainbow trout	Palti et al. 2009 [31]
Common carp	Xu et al. 2011 [32]
Pacific white shrimp	Yu et al. 2015 [179]
Asian seabass	Xia et al. 2010 [33]
Scallop	Zhang et al. 2011 [35]

### Transcriptome resources

Proper annotation of the genome sequences presents a challenge that can be at least partially overcome with transcriptome information. Specifically, gene models and gene structures need to be supported by experimental data; exon-intron borders need to be defined; alternatively spliced and differentially polyadenylated transcripts need to be identified and their translated proteins verified; and expression and function of the genes need to be studied. In addition to protein-coding genes, non-coding RNAs need to be identified and mechanisms of their target interactions need to be understood.

Large numbers of expressed sequence tag (EST) resources exist for major aquaculture species. As summarized in Table 8, almost a half million ESTs were generated for Atlantic salmon, over 350,000 for channel catfish, and almost 290,000 for rainbow trout. These EST resources are useful for the assembly of full length transcripts for genome annotation; however, with the advent of low-cost next generation sequencing technologies, transcriptomes are now more efficiently characterized with RNA-Seq.

**Table 8:** EST resources of selected aquaculture species (with >10,000 ESTs)

Species	Number of ESTs
Danio rerio (zebrafish)	1,488,275
Ciona intestinalis	1,205,674
Xenopus laevis (African clawed frog)	677,911
Oryzias latipes (Japanese medaka)	666,891
Salmo salar (Atlantic salmon)	498,245
Ictalurus punctatus (channel catfish)	354,516
Oncorhynchus mykiss (rainbow trout)	287,564
Morone saxatilis (striped bass)	230,151
Crassostrea gigas	206,388
Litopenaeus vannamei	161,248
Ictalurus furcatus	139,475
Oreochromis niloticus (Nile tilapia)	120,991
Petromyzon marinus (sea lamprey)	120,731
Sparus aurata	79,216

Zebrafish is included as a reference

Large RNA-Seq datasets have been generated by various institutions for important aquaculture species in the United States (https://www.ncbi.nlm.nih.gov/sra) to characterize differentially expressed genes in response to disease or stress in catfish [37–40], disease in salmon [41, 42] and to identify markers associated with growth, heat stress, and disease and tissue specificity in rainbow trout [43–46]. In striped bass, RNA-Seq studies focused on reproduction traits and egg quality [47–49], while in tilapia, they were conducted to identify genes responsive to alkalinity stress [50], salinity adaptation [51], and adaptation to low or high fat diets [52]. In yellow perch and bluegill, RNA sequencing of neo-males (perch), neo-females (bluegill), regular males and regular females is being conducted to investigate epigenomic modification of SSD and sex determination in fish (Wang, personal communication). RNA-Seq studies have also been conducted to characterize the Pacific oyster response to environmental stress (e.g., temperature, salinity, air exposure and heavy metals) [9, 53–55] and Ostreid herpesvirus [52]. In eastern oysters, RNA-Seq studies identified genes associated with osmoregulation [12], characterized the transcriptomic response to a bacterial pathogen [56], and revealed extensive expansion of gene families associated with innate immunity [15, 57]. In shrimp, genes associated with early development [58] and resistance to Taura syndrome virus (TSV) [59] have been identified via RNA-Seq analysis, and improved shrimp transcriptome were reported [60]. When coupled with genetic analysis such as bulk segregant analysis (e.g., [38, 43]), transcriptome analyses using RNA-Seq will enable the identification of candidate genes for important aquaculture traits.

Transcriptome resources also empower proteomics analysis [48, 61, 62]. Proteomics offers great promise for advancing our understanding of the functions of genes that underlie important production traits, however these methods rely on existing homologous protein-coding sequence databases, which remain incomplete for many non-model organisms, including important aquaculture species. Tandem mass spectrometry approaches in proteomics use these databases to identify protein fragments by mass spectrometry and thus require amino acid (or protein-coding nucleic acid) sequence information, optimally from the research organism under investigation. Thousands of different proteins have already been identified and measured with tandem mass spectrometry approaches to answer important questions about reproduction in striped bass and the closely related white perch, which serves as a research model [61–64]. A similar proteomic approach identified important proteins related to muscle atrophy in rainbow trout [65].

### Non-coding transcripts, regulation of genome expression, and epigenomics

Despite their importance in regulating gene expression, non-coding transcripts are much less understood than protein-coding transcripts in aquaculture species. Limited work has been conducted in this relatively new area of research. Among aquaculture species, most of the work on non-coding RNAs was conducted in rainbow trout. A few studies were devoted to identification of microRNAs and long non-coding RNAs [66–71]. In a number of cases, microRNAs were found to be associated with performance traits. For instance, a large number of microRNAs were differentially expressed between sexually mature and immature fish; in association with egg quality and muscle growth and quality [72–74]. In addition, differential expression of long non-coding RNAs studied in three genetic lines of rainbow trout identified important long-coding RNAs in response to infection with *Flavobacterium psychrophilum* [75].

In Atlantic salmon, several studies were conducted to characterize the microRNA repertoire. In one study, Bekaert et al. [76] identified 888 microRNA genes. In another study, Andreassen et al. [77] identified a total 180 distinct mature microRNAs, and found that many microRNAs were conserved across species, and a few microRNAs were expressed in a tissue-specific fashion. In another study, Kure et al. [78] found that 18 microRNAs were differentially expressed upon exposure to acidic aluminium-rich water.

Research on non-coding RNAs in catfish, striped bass, tilapia, oysters, and shrimp is limited. For instance, residue microRNA profiling was reported in catfish [79–81], tilapia [82], oysters [83, 84], and shrimp [85, 86]. However, now with the high quality reference genome sequences, it is expected that large numbers of projects will be conducted with aquaculture species in this area. This aligns very well with the FAANG (Functional Annotation of Animal Genomes) Project. As the importance and detailed operational protocols are well discussed in the white paper published in Genome Biology [87], we will not repeat them here, but this will be an important area for future research with aquaculture species as well, especially those with a well assembled reference genome sequence.

Genome scale analysis of epigenetic regulation have been conducted with oysters [88–93], Atlantic salmon [94], rainbow trout [95, 96], and tilapia [97], yellow perch, bluegill (Wang, personal communication) and additional projects are being initiated in several other major aquaculture species. Apparently, this is an area of active research, and functional annotation of non-protein coding genome elements is an important area. Again, this aligns well with those objectives of the FAANG Project [87].

### Performance traits, phenotypic variations, and QTL analysis

The practical purpose of aquaculture genomics and genetics studies is to reveal the genetic basis of performance and production traits, and to use such information for genetic enhancement programs. Domestication of most aquaculture species is still in the early stages, occurring over the last few decades, compared to other food animals and crops which have been domesticated over hundreds or even thousands of years. Because of this short history of domestication, aquaculture species still segregate considerable genetic variation among strains, lines, families and individuals.

Many aquaculture phenotypes are complex and quantitative in nature. Therefore, a major goal of aquaculture genetics research is to leverage genome information to predict complex phenotypes. In aquaculture species, QTL mapping and GWAS analysis are well-established procedures for correlating genetic and phenotypic variation; however additional work is required to identify specific genetic variants responsible for phenotypic variations. The identification of the causal SNPs or the genes underlining the performance traits is not only important for aquaculture applications, but also important for understanding the molecular mechanisms of phenotypic expression.

Progress with QTL/GWAS analysis has been greatly accelerated by the application of SNP arrays. Some examples of QTL mapping and GWAS analysis in aquaculture species are listed in Table 9. Most of the work has focused on disease resistance, growth traits, tolerance to stresses, and development or sexual maturity. Some of the best examples of QTL studies are from salmon research. For instance, the resistance against infectious pancreatic necrosis (IPN) virus was mapped to a major QTL that account for vast majority of phenotypic variance [98, 99], and further analysis identified the causal gene as epithelial cadherin [100, 101]. In catfish, QTL have been identified for a number of traits including disease resistance [38, 102, 103], heat stress [104], hypoxia tolerance [105], and head size [106]. In most of these cases, QTL were mapped within a region smaller than one million base pairs, allowing speculation of candidate genes, but fine mapping will be required to identify the specific causal genes. An interesting finding of these studies is the identification of functional hubs [102, 106] linking genes with roles in the same pathway. In addition, there appears to be a high level of evolutionary conservation of genes responsible for a number of traits in various species ranging across mammals, amphibians, and fishes. For instance, genes involved in the small GTPase pathway were found to affect head size and shape in catfish, frogs, mouse, and dogs [107]. Such discoveries open the possibility of comparative quantitative genomics.

**Table 9:** QTL studies in selected aquaculture species with major US aquaculture species in bold

Species	Traits	Reference
Arctic charr	Body weight and sexual maturation; Salinity tolerance	Küttner et al. 2011 [185]
		Norman et al. 2011 [186]
Asian seabass	Resistance against viral nervous necrosis disease	Liu et al. 2016 [187]
	Growth-related traits	Wang et al. 2006 [188]
	Omega-3 fatty acids	Xia et al. 2014 [189]
**Atlantic salmon**	**Growth traits and flesh colour**	**Baranski et al. 2010** [190]**; Tsai et al. 2014** [191]**; 2015** [192]**; Moen et al. 2009** [99]**; 2015**
	**Resistance against IPN**	[101]**; Houston et al. 2008** [98]**; 2010** [100]
	**Late sexual maturation**	**Gutierrez et al. 2014** [193]
	**Resistance to pancreas disease**	**Gonen et al. 2015** [194]
**Catfish**	**Columnaris disease resistance**	**Geng et al. 2015** [102]
	**ESC disease resistance**	**Wang et al. 2013** [38]**; Zhou et al. 2017** [103]
	**Hypoxia tolerance**	**Wang et al. 2016** [105]**;**
	**Heat stress**	**Jin et al. 2016** [104]
	**Head size**	**Geng et al. 2016** [106]
Common carp	Muscle fiber traits	Zhang et al. 2011 [195]
	Morphometric traits	Boulton et al. 2011 [196]
	Swimming ability	Laghari et al. 2014 [197]
**Eastern oyster**	**Disease resistance**	**Yu and Guo, 2006** [110]
European seabass	Growth, body weight	Louro et al. 2016 [198],
	Morphometric traits and stress response	Massault et al. 2010 [199]
**Pacific white shrimp**	**Growth parameters**	**Andriantahina et al. 2013** [178]
**Giant tiger prawn**	**Disease resistance and sex determination**	**Robinson et al. 2014** [200]
Japanese flounder	Vibrio anguillarum resistance	Wang et al. 2014 [201]
**Pacific oyster**	**Growth**	**Guo et al. 2012** [112]
	**Resistance against summer mortality**	**Sauvage et al. 2010** [202]
	**Viability**	**Plough & Hedgecock, 2011** [111]**; Plough et al. 2016** [113]
Gilthead seabream	Skeletal deformities	Negrín-Báez et al. 2015 [203]
	Sex determination and body growth	Loukovitis et al. 2011 [204]
	Resistance to fish pasteurellosis	Massault et al. 2011 [205]
**Rainbow trout**	**Growth related traits**	**Kocmarek et al. 2015** [206]**; Wringe at al., 2010** [207]**; Leder at al., 2006** [208]**; Easton et al. 2011** [209]**; Miller et al. 2012** [210]
	**Spawning time; development rate**	
	**Upper thermal tolerance**	**Perry et al. 2005** [211]
	**Whirling disease resistance**	**Baerwald et al. 2011** [212]
	**Bacterial cold water disease resistance**	**Vallejo et al. 2014** [107]**; Palti et al. 2015** [108]**; Liu et al. 2015** [109]**; Campbell et al. 2014** [213]
	**IHNV disease resistance**	**Rodriguez et al. 2004** [214]**; Campbell et al. 2014** [213]
	**Fillet yield**	**Gonzalez-Pena et al. 2016** [26]
	**Osmoregulation capacity**	**Le Bras et al. 2011** [215]
	**Response to crowding stress**	**Rexroad et al. 2013** [216]**; Liu et al. 2015** [217]
Turbot	Growth traits	Sánchez-Molano et al. 2011 [218]
	Aeromonas resistance	Rodríguez-Ramilo et al. 2011 [219]
	Resistance against Philasterides	Rodríguez‐Ramilo et al. 2013 [220]
	Resistance to viral haemorrhagic septicaemia	Rodríguez-Ramilo et al. 2014 [221]
**Tilapia**	**Growth traits**	**Liu et al. 2014** [222]**; Wang et al. 2015** [223]
	**Sex**	**Palaiokostas et al. 2015** [224]

Similarly, QTL have been identified for growth and reproductive traits, upper thermal tolerance, osmoregulation capacity, stress responses, and disease resistance in rainbow trout. Significant efforts have been devoted to the analysis of resistance to bacterial cold water disease (BCWD) [107–109]. QTL analysis and genome selection for BCWD resistance are facilitating significant genetic improvement for this trait in rainbow trout [3, 4].

Although QTL have been identified for disease resistance, viability and growth-related traits in eastern and Pacific oysters [110–113], low marker density limits QTL resolution. Candidate gene-based studies have led to the identification of variation in a serine protease inhibitor associated with *Perkinsus marinus*-resistance in the eastern oyster [114]. QTL analysis in tilapia, striped bass, and shrimp are at the early stages, but with the efficient genotyping systems, rapid progress is expected.

Aside from lack of genetic and genomic resources (e.g., inbred lines/families, sequenced genomes, efficient genotyping platforms) in some aquaculture species, several additional challenges face aquaculture researchers. First, unlike many livestock species where phenotypic and genotypic data can be collected on a large proportion of the cultured animals, phenotypic and genotypic data collection on the entire population of an aquaculture species is impossible. It is therefore essential that aquaculture geneticists understand QTL in all strains used in the industry, because a QTL present in one population may not be present in another. Second, fish and shellfish are outbred species with extremely large numbers of founders. Their high fecundities make QTL analysis within families extremely efficient, but whether the identified QTL are conserved across families, strains, and populations are unknown.

### Genome-based technologies and regulatory framework

A number of technologies, including polyploidization, gynogenesis, androgenesis, sex reversal, gamete cryopreservation, and gene transfer, are still very useful for aquaculture breeding programs. There are opportunities for enhancing these technologies by using genomic information. At the same time, genomic research has generated new technologies that can be used for genetic enhancement of aquaculture species, including marker-assisted selection (MAS), genome selection (GS), and genome editing.

Marker-assisted selection has been successfully used in aquaculture. The best example of MAS in an aquaculture species is selection for disease resistance in Japanese flounder. A microsatellite locus, Poli9-8TUF, was mapped near the major QTL for resistance to lymphocystis disease. Additional analysis indicated that the disease resistance was controlled by a single gene, and that the resistance allele was dominant. Based on the marker linkage information, Fuji et al. [115] developed a new population of Japanese flounder using MAS with the marker Poli9-8TUF. They selected a female homozygous for the favourable allele (B-favourable) and a male with a higher growth rate and good body shape, but without the resistant allele as parents. All the progeny are heterozygotes with the resistance allele and entirely resistant to lymphocystis disease, while the control group without B-favourable alleles showed incidences of 4.5 and 6.3% of mortality due to lymphocystis disease. These results clearly demonstrate that MAS is an efficient strategy for breeding [116].

Another good example of MAS is the selection of IPN resistance in Atlantic salmon. One major QTL was mapped to linkage group 21, which accounts for 29% and 83% of the phenotypic and genetic variances, respectively. Three microsatellite markers were tightly linked to the QTL, and these markers have been used for the selection of IPN resistance [99]. Recently, the gene responsible for IPN resistance was identified as a cadherin expressed in the epithelium where the protein binds to IPNV virions [101]. Marker-assisted selection allowed production of IPN-resistant salmon, leading to a 75% reduction in the number of IPN outbreaks in the salmon farming industry [101].

Sex identification using sex markers is a special case of MAS. Sex markers have been developed and used in quite a few aquaculture species, including common carp [117], tilapia [118], catfish [119], zhikong scallop [120], half-smooth tongue sole [121], white shrimp [122], kuruma prawn [123], yellowtail [124] and rainbow trout [125]. These sex-linked markers have been useful for the identification of sex without phenotypic data.

Recent advances in genome analysis including the availability of a large number of polymorphic markers, highly efficient genotyping platforms such as SNP arrays, and the application of next generation sequencing technologies, allowed mapping of dense markers across the entire genome, which in turn enables an estimation of the genetic merit of every chromosome fragment contributing variation in a population with phenotypic observations. Not only can the merit of every chromosomal segment be estimated, but also all the traits of interest can be estimated simultaneously. Whole genome selection is based on estimating the value of every chromosomal fragment contributing variation in a population with phenotypic observations (Training), and then the results of training are used to predict the merit of new animals (Testing) that are not included in the training dataset.

Genome selection was first proposed by Meuwissen et al. [126]. Since then it has gained tremendous attention in the animal genetics community. Compared with MAS, genomic selection uses the estimated effect of many loci across the entire genome at once, not just the small number of linked loci as done with MAS. Although genome selection has been successfully used in dairy cow and beef cattle and other livestock species [127], its use in aquaculture species has been limited to just a few species [128, 129]. In rainbow trout, genome selection was carried out for the selection of bacterial cold water disease [3]. In Atlantic salmon, genome selection was used to predict breeding values for resistance to sea lice [130]. Although demonstrated to be effective, genome selection has not been commercially applied in aquaculture species primarily due to financial limitations.

Supervised machine learning is similar in concept to whole genome selection using Training and Testing datasets, and includes Support Vector Machines (SVMs) and Artificial Neural Networks (ANNs). These are systems that can be trained to recognize certain data input patterns and then can be used to predict outcomes or classify data. Machine learning has been used to classify transcriptome and proteome data by pattern recognition (expression “fingerprinting”) in an analytical bioinformatics approach [49]. Expression patterns of genes and proteins can be modelled to identify the most important ones contributing to a trait or response. Machine learning ANNs have been used to analyze tens of thousands of expressed genes in microarray and RNA-Seq studies to show that the collective changes in the expression of 233 ovary genes (less than 2% of the genes measured) explained over 90% of the variation in striped bass embryo survival [47, 49]. These trained ANNs also predict, with a correct classification rate over 80%, which female striped bass will produce fertile or infertile eggs based on gene expression profiles of ovary tissues sampled prior to ovulation. Additionally, SVMs have been used to model the striped bass ovary proteome (355 proteins) and this system can predict the specific ovary growth stage with 83% accuracy based on quantitative tandem mass spectrometry data [61]. A portion of the plasma proteome (94 proteins) also has been similarly modelled to accurately predict gender of white perch [131]. Therefore, machine learning additionally poses a potential use as a diagnostic tool, for example in identifying those females that will produce poor quality eggs, or determining reproductive state or gender. Future applications of machine learning could include modelling genomic markers, such as SNPs, to identify those most important to a particular trait and then to predict the future performance of an individual based on the presence or absence of those SNP markers.

Genome editing refers to the ability to make specific changes at targeted genomic sites [132]. With the initial zinc finger nuclease (ZFN) technology developed in 1996, genome editing technologies have evolved and become more and more efficient, with the development of TALEN (transcription activator-like effector nucleases) and CRISPR/Cas9 (clustered regulatory interspaced short palindromic repeats). These new genome editing technologies overcome the disadvantages of ZFN technology and they have become very efficient for the modification of genomes. CRISPR/Cas9 has been demonstrated to be very efficient in zebrafish [133, 134], tilapia [135] and catfish (Liu, unpublished data). Mutation rates of 70–100% can be achieved with very low levels of mosacism in channel catfish [136].

Genome editing technologies can be used to introduce an immediate improvement in a phenotype in a single generation; hence, these technologies hold great promise for improving aquaculture. However, genetically modified organisms (GMO) have encountered low public acceptance, especially with aquaculture species. As demonstrated with the lengthy approval process of AquAdvantage transgenic Atlantic salmon, decades of time and millions of dollars were spent in coping with the regulatory issues ([137]; Hackett, 2016, personal communications). It could be argued that genome editing technologies differ from traditional gene transfer technologies because no foreign DNA is introduced. The scientific community must be proactive of research in the area of regulatory issues and public perception. Escaping the GMO label is possible with genome editing. For example, recently, the USDA decided that a CRISPR-modified mushroom can be cultured and sold without passing through the agency’s regulatory process [138].

### Leveraging Investments in Genomics through Integration with Germplasm Repositories

Rapid development and adaptation of genomic tools among various aquatic species is a double-edged sword in terms of how such tools may create genetic diversity and thereby limit industry options in the future. Evidence of such contractions have been demonstrated with livestock and in particular the Holstein cow and how gene banks can facilitate the alleviation of genetic bottlenecks [139, 140]. While genomic research continues to rapidly proceed among various aquaculture species, there are some major technological gaps preventing the aquaculture sector from securing and utilizing improved genetic resources. As shown in other life forms such as livestock species, there is a critical need to understand and acquire genetically diverse samples from all major aquatic species, cryopreserve those samples, and to present them in publically available databases for viewing of information about the sampled populations. Such information would include phenotypes, management system descriptors, environmental conditions, locality data, and comprehensive genomic information. The Animal-GRIN information system operated by USDA/ARS is designed in this manner and is publically accessible via the internet. Acquiring and integrating this wide range of data not only serves to make germplasm and tissue samples more useful in the present, it will also allow researchers to perform studies not foreseen today and to respond to future challenges such as disease outbreaks or losses of critical genetic diversity in cultured lines. Viewing of genetic resources (via germplasm) and its associated detailed information as a public resource serves to speed innovation, as well as to leverage the considerable investments being made in genomic research. In essence this affords us new and more cost-effective approaches to produce, maintain, and distribute genetic improvement across the breadth of cultured aquatic species [141].

To respond to these needs, there is a requirement to collect and cryogenically store gametes and tissues from a wide range of species that can be used by industry members and public researchers alike. Coupled with these samples should be the ability to store genomic information from publicly funded research, as well as from industry. Such an information system would link samples with genomic, phenotypic, locality (GIS-based), and environmental descriptors and make this information publically available through a user interface via the internet. Aquatic species researchers could use this resource for varied experimental purposes (e.g., of crossing spring and fall spawning populations) and for corrective mating. As such, the collection and curation of germplasm or tissue samples has value, just as does the determination of genomic information. It is the purposeful integration of these genetic and informational resources that provides a synergistic leveraging or expansion of value and potential utility. Indeed, the value of information or germplasm samples is directly magnified by their coupling or association in a comprehensive repository system.

### Future research priorities

Economically important aquaculture species are a diverse group of organisms and research priorities vary depending on the unique biology of each species. Although fishes are the most diverse vertebrate group, aquacultured teleosts are similar enough phylogenetically and biologically that they can follow a similar research program. Invertebrates are not as uniform, and may each have special properties that require different approaches.

**Fig. 1:**
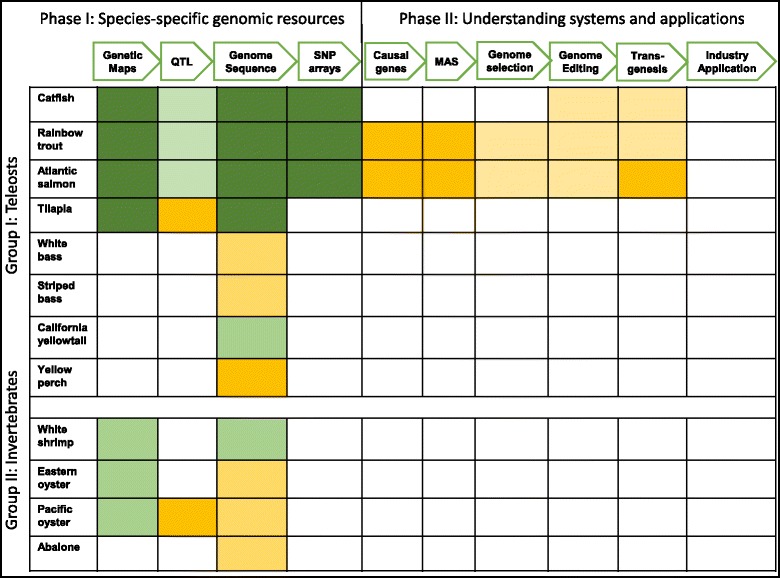
Schematic presentation of the goals and current status of aquaculture genomics and genetics research. The major aquaculture species in the United States are grouped into teleost fish and invertebrate species, with the species names listed in the first column. Major milestones of research goals are listed in the first row, while current status for each species is indicated in the appropriate cells with various colors: *Dark green*: good status; *light green*, outstanding progress has been made, but additional work still needed; *dark yellow*: significant progress has been made, but significant amount of additional work still needed; *light yellow*, some progress has been made

The research tasks needed to develop a program of genetic enhancement in aquaculture species can be divided into two phases (Fig. 1). The first phase, development of species-specific genomic resources, is the one that has been pursued for the major aquaculture species over the past 20 years. It includes the development of genetic and physical maps, annotated genome sequences, and platforms for high-throughput genotyping. Some species (e.g., catfish, tilapia, rainbow trout, and salmon) may be nearing the completion of Phase I. Other species are just beginning this phase, hopefully benefitting from the experience of other species, and taking shortcuts available with new technologies. From the perspective of genetics and breeding, generation of complete sets of heritabilities and genetic correlations is also needed.

Within Phase I, we can make a distinction between development of resources and application of resources to commercial aquaculture. For instance, genetic maps enable QTL/MAS, and genome sequences enable genomic selection. Application of MAS/genomic selection is generally beyond the scope and funding of the academic laboratories that have participated in the development of the genomic tools that enable it, although not beyond the scope and mission of government laboratories and academic laboratories that take on genetic stock enhancement for smaller or regional aquaculture species.

In Phase II, the genomic resources developed in Phase I can be used to develop a functional understanding of animal systems. As an example, a number of laboratories are working to develop an understanding of the gene regulatory network underlying sex determination in fishes. The research program involves not only RNA-Seq to characterize patterns of gene expression in the developing gonad, but also CRISPR modifications to test linkages in the gene regulatory network model. It should be possible to develop an understanding of the gene network underlying sex determination that will be broadly applicable among aquaculture species. Similar research programs are underway to understand the genetic basis of other important traits including growth, disease resistance, etc. In each case, the goal is to develop an understanding of animal systems that can be easily transferred to related species.

When this more detailed understanding of animal systems is complete, it will become possible to make specific genetic modifications (e.g., using CRISPR) to improve animals for commercial production. The safety and effectiveness of such modifications are important topics for research, but commercial application of these technologies will require stable business models and well established regulatory frameworks that ensure the safe application of these technologies to the species being targeted, the public, and the environment.

The current status of breeding technologies in US commercial aquaculture is summarized in Table 10. With significant differences in the structures of the aquaculture industries among species, practical strategies suitable to specific situations must be developed. In addition, development of comprehensive germplasm repositories will ensure protection of valuable genetic resources of aquaculture species and the investments made in developing them.

**Table 10:** Current status of breeding technologies in U.S. commercial aquaculture

Species	Status
Catfish	Private sector efforts to conduct genetic enhancement programs appear to have been successful, but the private sector has not made a great effort in genetics and breeding. Currently, some on-farm selection is practiced, but not in a very controlled manner. Genetic improvement is primarily conducted by public sector research programs, which has resulted in 7 releases to the industry of varying impacts. Most of these fish populations were developed by mass selection and in some cases family selection with the most emphasis on growth rate. Advanced genomic tools and technologies are available but have yet to be implemented by industry.
	The industry has widely adopted the channel female x blue male interspecific catfish hybrid which demonstrates significantly greater performance for numerous traits in comparison to the traditionally grown channel catfish with hybrids now comprising 60–70% of the industry. The vast majority of hybrids are produced with a single line of blue catfish.
Atlantic salmon	Private sector breeding is integrated with a publicly funded research program. Genetic improvement is based on quantitative genetics to improve growth, fillet quality and disease traits. Due to international interest in this species advanced genome tools and technologies are widely available, their implementation in the U.S. was recently initiated in a public/private partnership with efforts to incorporate MAS for sea lice resistance.
	In 2015 the AquAdvantage Salmon was approved for sale in the U.S. by FDA, however it is expected to reach the marketplace in 2017.
Rainbow trout	Public sector breeding programs utilize quantitative genetics to select for growth performance and disease resistance in all-female populations. Chromosome set manipulation is used to provide all-female triploids for net pen operations that require sterile fish; they are also valued for their superior growth characteristics at larger sizes.
	Publically funded research programs have released germplasm improved for growth and disease resistance characteristics. Advanced genome tools and technologies are widely available and have been implemented into the private sector. Proof of concept studies for genomic selection for disease resistance in a research population have motivated initial implementation in a commercial breeding population.
Tilapia	Private sector family based breeding for Nile tilapia for improved growth, yield and disease resistance is enhanced through publicly funded research programs. Although genome tools and technologies are available, they have not yet been implemented by the private sector.
Striped bass	Private sector fingerling producers incorporate germplasm from wild caught and captive (domestic) populations. Significant genetic improvement has been achieved through the production of hybrids created primarily by crossing domestic striped bass males x domestic or wild caught white bass females, with parental species improvement achieved primarily via mass selection techniques. Genomic technologies are under development and have not yet incorporated into commercial breeding, although domestic striped bass and white bass are available through a publically funded research program.
Oysters	The Pacific oyster industry is supported through public and private programs for ploidy manipulation, family-based selection and crossbreeding. Polyploid and improved broodstocks are widely used by the U.S. West Coast industry. Genetic improvement of the eastern oyster is publically funded. For much of the past 40 years, improvements in eastern oyster growth and survival have been realized using mass-selection techniques; however, there has been a recent shift toward applying quantitative genetics and ploidy manipulation to enhance production traits. Broodstock from these breeding programs are widely used by the private sector in the Northeast and Mid-Atlantic. Genome tools for both oyster species are coming online, but have not yet been implemented.
Shrimp	Shrimp breeders in the public and private sector selectively breed to produce specific pathogen resistant shrimp.

## Conclusions

Based on the current status, trends, and industry needs of aquaculture genomics, genetics and breeding research, the following areas of research need to be priorities:

Phase I goals for each species

- Highly contiguous and complete genome sequence
- Full annotation of the genome sequence, including functional (genome to phenome) studies
- Identification of genetic variants in different broodstocks, and their relationship to performance traits
- Development of systems for high-throughput genotyping
- Anchoring of the genome sequence to genetic maps
- Identification of QTL for performance and production traits
- Bioinformatic capabilities to manage these data
- Training the next generation of aquaculture breeders
- Establishment of high-throughput cryopreservation protocols and pathways for aquaculture species

Phase II goals for each group of species

- Proof of concept demonstrations which apply genome technologies to improve production efficiency, production sustainability, animal welfare and/or product quality in the commercial sector
- Development of standardized measures of organismal phenotypes
- Understanding epigenetic effects that contribute to variation in gene expression
- Validation of QTL, identification of the causative genetic variants underlying variations in performance, and determine the mechanisms of actions
- Determine general and specific combining abilities in both intraspecific and interspecific systems
- Marker-assisted selection and genome selection for production traits
- Characterization of the gene regulatory networks underlying phenotypic traits important to commercial aquaculture production
- Determine the genomic basis of heterosis and genomic predictors of heterosis
- Identification of conserved regulatory mechanisms and pathways for growth, feed conversion efficiency, disease resistance, stress tolerance, sex and other traits among aquaculture species
- Development and application of gene editing technologies and the associated regulatory frameworks, first for basic research, and eventually for commercial production
- Development of tools that can be easily used by the industry
- Industry applications of genome technologies
- Establishment of a comprehensive germplasm repository system to protect, maintain and distribute genetic resources developed through genomic technologies

## Abbreviations

ANN: Artificial neural networks
CRISPR: Clustered regulatory interspaced short palindromic repeats
ESD: Environmental sex determination
EST: Expressed sequence tag
FAANG: Functional Annotation of Animal Genomes
GMO: Genetically modified organisms
GS: Genome selection
GSD: Genetic sex determination
GWAS: Genome-wide association studies
IPN: Infectious pancreatic necrosis
IPNV: Infectious pancreatic necrosis virus
MAS: Marker-assisted selection
NRSP-8: National Research Support Project 8
QTL: Quantitative trait loci
SNP: Single nucleotide polymorphism
SPF: Specific pathogen-free
SSD: Sexual size dimorphism
SVM: Support vector machines
TALEN: Transcription activator-like effector nucleases
ZFN: Zinc finger nuclease
