# Erratum to: Aquaculture genomics, genetics and breeding in the United States: current status, challenges, and priorities for future research

**DOI:** 10.1186/s12864-017-3614-9

**Published:** 2017-03-16

**Authors:** The Aquaculture Genomics, Genetics and Breeding Workshop, Hisham Abdelrahman, Mohamed ElHady, Acacia Alcivar-Warren, Standish Allen, Rafet Al-Tobasei, Lisui Bao, Ben Beck, Harvey Blackburn, Brian Bosworth, John Buchanan, Jesse Chappell, William Daniels, Sheng Dong, Rex Dunham, Evan Durland, Ahmed Elaswad, Marta Gomez-Chiarri, Kamal Gosh, Ximing Guo, Perry Hackett, Terry Hanson, Dennis Hedgecock, Tiffany Howard, Leigh Holland, Molly Jackson, Yulin Jin, Karim Khalil, Thomas Kocher, Tim Leeds, Ning Li, Lauren Lindsey, Shikai Liu, Zhanjiang Liu, Kyle Martin, Romi Novriadi, Ramjie Odin, Yniv Palti, Eric Peatman, Dina Proestou, Guyu Qin, Benjamin Reading, Caird Rexroad, Steven Roberts, Mohamed Salem, Andrew Severin, Huitong Shi, Craig Shoemaker, Sheila Stiles, Suxu Tan, Kathy F. J. Tang, Wilawan Thongda, Terrence Tiersch, Joseph Tomasso, Wendy Tri Prabowo, Roger Vallejo, Hein van der Steen, Khoi Vo, Geoff Waldbieser, Hanping Wang, Xiaozhu Wang, Jianhai Xiang, Yujia Yang, Roger Yant, Zihao Yuan, Qifan Zeng, Tao Zhou

**Affiliations:** 1 School of Fisheries, Aquaculture and Aquatic Sciences, Auburn University, Auburn, AL 36849 USA; 2 Department of Biological Sciences, Auburn University, Auburn, AL 36849 USA; 3 Environmental Genomics Inc., P. O. Box 196, Southborough, MA 01772-1801 USA; 4 Aquaculture Genetics & Breeding Technology Center, Virginia Institute of Marine Science, Gloucester Point, VA 23062 USA; 5 Department of Biology, Middle Tennessee State University, Murfreesboro, TN 37132 USA; 6 Aquatic Animal Health Research Unit, USDA-ARS, 990 Wire Road, Auburn, AL 36832 USA; 7 USDA-ARS-NL Wheat & Corn Collections at a Glance GRP, National Animal Germplasm Program, 1111 S. Mason St., Fort Collins, CO 80521-4500 USA; 8 USDA-ARS/CGRU, 141 Experimental Station Road, Stoneville, MS 38701 USA; 9 Center for Aquaculture Technologies, 8395 Camino Santa Fe, Suite E, San Diego, CA 92121 USA; 10 Department of Fisheries and Wildlife, Oregon State University, Corvallis, OR 97331 USA; 11 Department of Fisheries, Animal & Veterinary Science, 134 Woodward Hall, 9 East Alumni Avenue, Kingston, RI 02881 USA; 12 Haskin Shellfish Research Laboratory, Department of Marine and Coastal Sciences, Rutgers University, 6959 Miller Avenue, Port Norris, NJ 08349 USA; 13 Department of Genetics, Cell Biology and Development, 5-108 MCB, 420 Washington Avenue SE, Minneapolis, MN 55455 USA; 14 Department of Biological Sciences, University of Southern California, Los Angeles, CA 90089-0371 USA; 15 Taylor Shellfish Farms, 130 SE Lynch RD, Shelton, WA 98584 USA; 16 Department of Biology, University of Maryland, 2132 Biosciences Research Building, College Park, MD 20742 USA; 17 National Center for Cool and Cold Water Aquaculture, Agricultural Research Service, United States Department of Agriculture, Kearneysville, WV 25430 USA; 18 Troutlodge, 27090 Us Highway 12, Naches, WA 98937 USA; 19 USDA ARS NEA NCWMAC Shellfish Genetics at the University Rhode Island, 469 CBLS, 120 Flagg Road, Kingston, RI 02881 USA; 20 Department of Applied Ecology, North Carolina State University, Raleigh, NC 27695-7617 USA; 21 USDA ARS Office of National Programs, George Washington Carver Center Room 4-2106, 5601 Sunnyside Avenue, Beltsville, MD 20705 USA; 22 School of Aquatic and Fishery Sciences, University of Washington, Seattle, WA 98105 USA; 23 Genome Informatics Facility, Office of Biotechnology, Iowa State University, Ames, IA 50011 USA; 24 USDOC/NOAA, National Marine Fisheries, NEFSC, Milford Laboratory, Milford, Connectcut 06460 USA; 25 School of Animal and Comparative Biomedical Sciences, University of Arizona, Tucson, AZ 85721 USA; 26 Aquatic Germplasm and Genetic Resources Center, School of Renewable Natural Resources, Louisiana State University Agricultural Center, Baton Rouge, LA 70820 USA; 27 Stonebridge breeding Ltd, Gate House, Abbotswood, Evesham, WR11 4NS UK; 28 Aquaculture Genetics and Breeding Laboratory, The Ohio State University South Centers, Piketon, OH 45661 USA; 29 Key Laboratory of Experimental Marine Biology, Institute of Oceanology, Chinese Academy of Sciences, Qingdao, 266071 China; 30 Hybrid Catfish Company, 1233 Montgomery Drive, Inverness, MS 38753 USA

## Erratum

In the version of this article that was originally published [1] there was an error with the author name “Karim Khalil” as it was incorrectly put as “Karim Kahlil”. The original article was corrected.
